# Knowledge, Attitude, and Practices towards Dengue Fever among University Students of Dhaka City, Bangladesh

**DOI:** 10.3390/ijerph19074023

**Published:** 2022-03-28

**Authors:** Md Mostafizur Rahman, Saadmaan Jubayer Khan, Kamrun Nahar Tanni, Tuly Roy, Musabber Ali Chisty, Md. Rakibul Islam, Md. Alim Al Raji Rumi, Mohammed Sadman Sakib, Masrur Abdul Quader, Md. Nafee-Ul-Islam Bhuiyan, Farzana Rahman, Edris Alam, Abu Reza Md. Towfiqul Islam

**Affiliations:** 1 Department of Disaster and Human Security Management, Faculty of Arts and Social Sciences, Bangladesh University of Professionals, Mirpur Cantonment, Dhaka 1216, Bangladesh; mostafizur@bup.edu.bd (M.M.R.); saadmaan.jubayer@bup.edu.bd (S.J.K.); kamrun.nahar3103@gmail.com (K.N.T.); roy.tuly04@gmail.com (T.R.); rakib.rahid3005@gmail.com (M.R.I.); rahinrumi5@gmail.com (M.A.A.R.R.); sakibmhs@gmail.com (M.S.S.); maq9811@gmail.com (M.A.Q.); Nafee.bhuiyan.08@gmail.com (M.N.-U.-I.B.); 2 Institute of Disaster Management and Vulnerability Studies, University of Dhaka, Dhaka 1000, Bangladesh; musabber.chisty@du.ac.bd; 3 Department of Computer Science and Engineering, Independent University, Dhaka 1212, Bangladesh; farzana.rahman@iub.edu.bd; 4 Faculty of Resilience, Rabdan Academy, Abu Dhabi P.O. Box 22401, United Arab Emirates; ealam@ra.ac.ae; 5 Department of Geography and Environmental Studies, University of Chittagong, Chittagong 4331, Bangladesh; 6 Department of Disaster Management, Begum Rokeya University, Rangpur 5400, Bangladesh

**Keywords:** dengue, infectious disease, university students, urban planning, Dhaka city

## Abstract

Dhaka has become the worst affected city in Bangladesh regarding dengue fever (DF). A large number of university students are residing in this city with a high DF risk. This cross-sectional study was conducted to assess the DF status and responses among these students through their Knowledge, Attitude, and Practices (KAP) survey. A total of 625 students participated in an online self-reported survey. Statistical analyses were performed to assess the status and KAP regarding DF. University students from the city perceived their living places as moderately safe (45.28%) against DF, whereas about 20% reported their DF infection history. Some of these students had exemplary DF knowledge (66.72%), attitude (89.28%), and practices (68.32%). However, many of them were also observed with a lack of knowledge about this disease’s infectious behavior, recognizing *Aedes* mosquito breeding sites, multiple infection cases, and the risk of DF viral infection during pregnancy. Fair correlations (*p* < 0.001) were determined in the KAP domain. Gender, residential unit, major, and dengue-relevant subjects were found to be significant predictors (*p* < 0.05) of KAP level in the univariate analysis. Major subject and residential units remained significant predictors of overall KAP level in further multiple analysis. This study revealed the urgency of infectious disease-related subjects and the relevant demonstration into the university curriculum. The study’s findings can assist the university, government and non-governmental organizations, and the health and social workers to prepare a comprehensive dengue response and preparedness plan.

## 1. Introduction

Around 70% of dengue cases have occurred in the Asian region [1]. The south Asian region is a hotspot for various infectious diseases, such as dengue fever (DF) [2]. Bangladesh experienced the rapid rise of dengue cases in 2019 [3,4]. It was first documented as “Dhaka fever” in Bangladesh [2]. In the 2019 outbreak, Dhaka alone accounted for more than half of the country’s total dengue cases [5]. Prior study has revealed that the widespread DF cases in 2019 across the country were sparked by Dhaka city [3]. The major DF outbreak tended to be confined in this city [6]. The pertinent dengue cases since the year 2000 made DF a burden for the city. It has also been predicted that the future major DF outbreaks would be dominant within this city [6,7]. Besides, climate change, population density, rapid unplanned urbanization, and neglectful behavior towards DF could aggravate the current condition [4,8]. In light of these factors, Bangladesh’s national and local governments as well as other stakeholders and city inhabitants must prepare and respond effectively to stem the dengue outbreak’s spread.

There is no prophylaxis or effective vaccine for DF [9]. Health behaviors, such as adhering to authentic knowledge, maintaining a positive attitude, and following proper procedures, may only help reduce DF [10]. Individual and community behavioral changes and proper governmental support for DF can help reduce the rising dengue incidence in Dhaka and, as a result, across the country. One study performed in Dhaka, Bangladesh, where the current research was conducted, found that hospitalized patients have a high level of knowledge of dengue and a favorable attitude towards dengue-prevention methods [11]. Another study examined KAP among university students and found a statistically significant relationship between climatic change awareness and the DF KAP level in Bangladesh [8]. One study also conducted KAP survey regarding DF among university students of Bangladesh [12]. However, lack of study was found regarding DF among university students of Dhaka city, where the country’s worst DF cases were found.

Bangladesh is a land of natural catastrophic events [13,14] due to the geographical location and climate change, where infectious diseases have also become prevalent [4,15,16,17,18]. Dhaka, the capital city, serves as the focal point for all major activities in Bangladesh [3]. It is also one of the most densely populated areas of the world [19]. However, this megacity faces several challenges, including rapid development, migration from other parts of the country, and inadequate housing management for such a large population. It has also experienced the worst outbreaks of DF and COVID-19 [3,18].

University students are a critical group of learners in any community because they have access to authentic knowledge. The authority can easily reach them through universities or different social and digital media platforms [20,21]. If these students bear enough knowledge and positive attitudes, they can transfer it to their community. In collaboration with university students, the community can then translate knowledge and attitudes about DF into preventative practices. Thus, students can act as a crucial hub for community preparedness. Dhaka city is home to many government-funded public and private universities, with one per 5.38 square kilometers [22]. A massive number of university students live in this city. These students have high DF risk, like other people living in the city. Inadequate university premises and infrastructures can be a hotspot for DF [8]. These students also move across the country for educational and family purposes. As a result, they may act as carriers of the DF virus or as trusted media, disseminating authentic knowledge, positive attitudes, and preventive practices throughout the country. This study intended to evaluate the DF status and responses among these university students through the KAP (knowledge, attitude, and practice) survey model. The outcome of our study can assist the government and non-governmental organizations, university authorities, and health and social workers in fostering holistic DF preparedness and response for university students of the city, which can ultimately contribute to reducing the DF outbreak across the country. It can also be integrated into the strategic plan to control infectious disease outbreaks, a fundamental issue for the national and international community.

## 2. Materials and Methods

### 2.1. Conceptual Framework

We used the KAP model, which has already been successfully applied to evaluate the DF among university students [8,23,24]. However, none focused specifically on the status of university students in Dhaka city. This model was developed for family planning and demographic research in the 1950s [25,26]. Since then, it has been widely used to assess participants’ knowledge, beliefs, and actions on a given subject, most notably public health, in a social setting and research [17,21,27,28]. The advantages of this model are that it is simple to perform and that interpretations are reasonably straightforward with a brief presentation [29]. Additionally, it is claimed that this model demonstrates the connection between participants’ knowledge, attitudes, and practices [30]. As a result, it may assess the gap in the community’s preparedness for a disease by addressing the KAP level. The outcome may be utilized to create strategies aimed at increasing behavioral improvements [31].

### 2.2. Study Design and Ethical Issues

This cross-sectional study designed a self-reported online survey to evaluate the dengue responses through a KAP survey among university students of Dhaka city, Bangladesh. The study area was divided based on the city corporation area: Dhaka South City Corporation and Dhaka North City Corporation. This study was a part of an approved research project (Ref. 343/2019) from the Research Ethics Committee of Bangladesh University of Professionals, Dhaka, Bangladesh. This study contained no more than minimal risk. It has maintained all associated ethical issues. The cover page of the questionnaire clearly described the objective of the survey and the confidentiality of the responses. If respondents agreed, they were requested to participate in the survey. Online consents were also sought. Respondents were not granted any incentive. They were able to withdraw the questionnaire fill-up any time. Please check Supplementary File S1.

### 2.3. Survey Instrument and Data Collection

The final structured questionnaire (in both native Bengali and English language) was developed in Google Form after conducting literature reviews, discussing with experts, undertaking a pilot survey, and maintaining cultural appropriateness [8,28,32,33]. It had three sections with mandatory response items: socio-demographic and academic information, individual perception on the safety of living place against DF and their dengue infection history, and the final KAP section. A 5-point Likert scale (very safe, safe, moderately safe, unsafe, and very unsafe) was used for examining the students’ safety perception about their living place against DF. A simple question, “How safe do you think your current place is from dengue?” was in this section. In case of dengue infection history (if they were infected), Yes/No/Maybe options were available. A total 2of 8 closed-ended questions were in the KAP section. Twelve questions were for knowledge (0–1 score range, 0.5 for Maybe), eight for attitude (5-point Likert scale-based questions: Strongly agree, agree, neutral, disagree and strongly disagree), and eight (Yes/No) for the preventive practices. We calculated the Cronbach’s alpha (Equation (1)) value in pilot survey data, which showed the value as >0.70, >0.80, and >0.80 for KAP section. We reran the final data, and Cronbach’s alpha was calculated as an accepted reliable value of 0.69, 0.76, and 0.87 for knowledge, attitude, and practice, respectively, to assess the KAP section’s internal consistency [34,35,36].

(1) $$\alpha =\frac{N.\bar{c}}{\bar{v}+\left(N-1\right).\bar{c}}$$

where, *N* = number of items, $\bar{c}$ = average covariance between item-pairs, and $\bar{v}$ = average variance.

The convenience sampling technique was followed for data collection. Convenience sampling is a type of non-probability sampling technique that is commonly employed in clinical research. Typically, this sampling technique selects clinical cases or participants from an easily accessible location, medical records database, internet site, or customer-membership list [37]. A group of university students were recruited based on their research experience and then instructed to administer the online questionnaire from August to December 2020 through Google classroom, Facebook, WhatsApp, and other available digital media. In August 2019, a vast number of dengue cases were recorded in Dhaka city [3,5,38]. This perception-based study required 384 sample units (95% Confidence Intervals) following Morgan’s table [39]. We approached approximately 750 university students of Dhaka city, where 625 of them were considered for final analysis. Thus, the response rate of the survey was 83.33%. Data collection was carefully monitored and double-checked in online Google form on a regular basis. It was then transferred to Microsoft excel format for further analysis.

### 2.4. Data Management and Analysis

Python (version 2.7; Beaverton, OR 97008, USA), RStudio (version 1.2.5042; Boston, MA, USA), and “R” programming language (version 3.6.3; Vienna, Austria) were used for data management and statistical analysis [40,41]. Shapiro–Wilk and Kolmogorov–Smirnov tests were performed to check the normality of numeric data. Data were not normally distributed. Descriptive statistical analyses (frequency and percentage for non-continuous data, the mean and standard deviation for continuous data) were calculated. Non-parametric Kruskal–Wallis or Mann–Whitney U tests were performed (where appropriate) to examine the association of socio-demographic and academic information with the safety perception and dengue infection history. Post-hoc analysis (Dunn’s test) was carried out with Bonferroni correction to calculate the adjusted *p*-value.

Spearman’s rank correlation was conducted to determine the correlation in the KAP domain. We calculated 80% cut-off scores (after summing the KAP section’s score) to categorize the “Good” and “Poor” levels in the KAP domain [42]. Univariate logistic regression analysis was performed to determine the predictors of KAP level. The multiple logistic regression analysis (where *p* < 0.25 significant interaction was observed in univariate analysis) was also performed [33]. Odds ratios (ORs), adjusted odds ratios (aORs), and 95% confidence intervals were calculated in the logistic regression models. All statistical analyses were performed at 0.05 α level.

## 3. Results

### 3.1. Socio-demographic and Academic Information

Table 1 presents the socio-demographic and academic information of participating university students from Dhaka city. In all, 51.04% were female, and 48.96% were male among 625 participants. These students have an age range of 18–30 years. A total of 80.64% were with their families, and 64.64% were from the Dhaka North City Corporation area. Many of them lived in high-rise building units (54.40%) and low-rise building units (40.32%). Most of the participated students were from public universities (86.40%). We only considered 1st–4th year and Master’s level students. In the case of a major, students were from diversified fields. However, most students (60.64%) did not have any dengue-relevant subject in their university curriculum.

### 3.2. Safety Perception and Dengue Infection History

The majority of the respondents perceived moderately safe places (*n* = 283, 45.28%) against DF followed by safe (*n* = 172, 27.52%) and unsafe (*n* = 108, 17.28%) perceptions. Many of the research participants confirmed (*n* = 117, 18.72%) that they had previously contracted DF, whereas the majority (*n* = 494, 79.04%) did not. Some individuals (*n* = 14, 2.24%) expressed skepticism regarding their infection.

Table 1 also illustrates socio-demographic and academic profiles associated with their safety perception and dengue infection history. A significant association was demonstrated between respondents’ university year and their dengue infection history. Post-hoc analysis identified that the Master’s level students reported significantly more dengue infection history than the first-year students. A significant association was also observed in the case of major with the living place’s safety perception against DF. Post-hoc analysis identified this association when comparing the business studies and science and engineering major students (perceived more safety) with the students from security and strategic studies.

### 3.3. Knowledge regarding Dengue Fever

Table 2 summarizes the DF-relevant knowledge among the participants. The majority of the participated students (>80%) positively reported the fatal effect of DF, common symptoms of this disease, *Aedes* mosquito type (female) responsible for dengue virus transmission, phenotypic recognition (stripe on the body) of this mosquito, and keeping the surrounding areas clean as a vital way to reduce this mosquito. Conversely, many of the research participants (>25%) were also unable to answer the questions regarding infectious behavior of this disease, human-vector (mosquito) contact as the main cause of the dengue virus infection (many answered the human-human contact), mosquito breeding sites, viral transmission from mother to the fetus, and multiple infections for the same person.

### 3.4. Attitude towards Dengue Fever

Table 3 shows the attitudes of the study participants towards DF. The majority of them strongly agreed (>50%) or agreed (>30%) that they should clean *Aedes* mosquito breeding sites, that only chemical fogging is not enough to control DF, and that monitoring DF situation and the immediate checkup of family members is required if any DF symptoms appear. However, many of them also agreed that they are responsible for ensuring *Aedes* mosquito-free housing area where regular activities and strong community commitments are required to participate in the DF control public activity.

### 3.5. Preventive Practices towards Dengue Fever

Table 4 presents that most of the study participants (>80%) demonstrated the preventive practices towards DF, such as using mosquito-controlling tools and liquids, covering and cleaning the water storage containers and pots, and visiting the hospital for dengue virus test if any symptoms appear. However, many of them did not follow other preventive practices, such as communication with the authority for fogging (>70%), monitoring for the presence of *Aedes* mosquito eggs and larvae (>45%), and following the updated information from trusted sources (>20%).

### 3.6. Association in KAP Domain

Significant positive correlations (*p* < 0.001) between knowledge and attitude, knowledge and practices, attitude and practices were determined (Table 5). Linear regression analysis identified knowledge and attitude as significant predictors (*p* < 0.001) of practices towards DF. ANOVA test also confirmed the significance of the regression model (*p* < 0.001, adjusted R^2^ = 0.145).

### 3.7. KAP Domain Level towards Dengue Fever

Many participated university students demonstrated the good knowledge level (*n* = 417, 66.72%), good attitude level (*n* = 558, 89.28%), good preventive practice level (*n* = 427, 68.32%), and good overall KAP level (*n* = 478, 76.48%). Table 6 summarizes the univariate analysis results. A significant association was found between the gender and their attitude and practice level. Female students reported increased odds of having a good attitude (OR: 1.74; 95% CI: 1.04–2.96) and a good practice (OR: 1.43; 95% CI: 1.02–2.00) compared to the male counterparts. Similarly, increased odds of having a good knowledge (OR: 4.79; 95% CI: 2.30–10.41) and a good overall KAP (OR: 3.76; 95% CI: 1.80–7.86) were found when the study population was from high-rise units compared to that of the mixed-unit participants. The low-rise unit respondents also displayed increased odds of having good knowledge (OR: 2.84; 95% CI: 1.36–6.21) and good overall KAP (OR: 2.82; 95% CI: 1.34–5.94). First-year students demonstrated increased odds of having a good attitude (OR: 4.96; 95% CI: 1.44–17.16) compared to the Master’s level students. Furthermore, business studies major students showed decreased odds of having a good knowledge (OR: 0.40; 95% CI: 0.22–0.70), a good attitude (OR: 0.32; 95% CI: 0.12–0.75), a good practice (OR: 0.52; 95% CI: 0.28–0.93), and a good overall KAP (OR: 0.25; 95% CI: 0.12–0.51) compared to other miscellaneous major students. Security and strategic studies major students also had decreased odds of having a good practice (OR: 0.29; 95% CI: 0.16–0.52) and a good overall KAP (OR: 0.27; 95% CI: 0.12–0.54). Students majoring in arts and social sciences reported decreased odds of having a good overall KAP (OR: 0.44; 95% CI: 0.20–0.91). A significant association was demonstrated between the dengue-relevant subject in the university curriculum and knowledge level. Students who confirmed a subject covering knowledge regarding DF reported increased odds of having a good knowledge (OR: 1.71; 95% CI: 1.02–2.98) compared to those who did not have it.

Table 7 shows the multiple analyses results. Adjusted odds ratios (aORs) were produced using multiple regression, which employed several independent variables. aORs take into account other predictor variables in a model. After screening the associated factors in univariate analyses, only the significant factors that satisfied *p* < 0.25 were considered for multiple analyses. The residential unit type and major remained significant predictors of preventive practices and overall KAP. High-rise (aOR: 3.38; 95% CI: 1.56–7.31) and low-rise unit residents (aOR: 2.63; 95% CI: 1.20–5.75) had a higher odds of good overall KAP compared to the mixed unit residents. Furthermore, the major field of study also demonstrated significant predictors of preventive practices and overall KAP. Business studies (aOR: 0.24; 95% CI: 0.11–0.54) and arts and social sciences (aOR: 0.42; 95% CI: 0.19–0.87) major students showed significantly decreased odds of having a good overall KAP compared to other miscellaneous major students. Students were also found to have lower odds of good practice (aOR: 0.31; 95% CI: 0.16–0.56) and good overall KAP (aOR: 0.26; 95% CI: 0.12–0.54) if they were from security and strategic studies majors.

## 4. Discussion

University students have become a substantial part of Dhaka city, where they have a high risk of DF [11,43]. These students typically originated from different parts of the country but live in the capital for better education and job opportunity. Therefore, fast detection and intervention among these learners and commuters can strengthen the whole country’s dengue outbreak control strategy. In addition, they can act as a hub between the authority and the other social groups [8,20,21]. Nevertheless, there was still no notable study regarding KAP aspects towards DF among university students of Dhaka city despite the frequent outbreak.

Our findings revealed that few of the participated students learned about DF in the university curriculum. Universities could contribute to health promotion where students could play a crucial role in preparing their surrounding community [23]. They could also integrate infectious diseases relevant subjects into their curriculum. University Grants Commission (UGC) of Bangladesh could also play a substantial role in this aspect. Moreover, the COVID-19 pandemic situation has been urged for this type of intervention.

### 4.1. Determinants of Dengue Fever Status

This study demonstrated that the students were concerned about their place’s safety against DF. The city dwellers already have experienced numerous types of hazard [44,45], and the COVID-19 pandemic [18,21] might trigger safety concerns among the students. The security and strategic studies major students perceived less confidence in the safety of their current place against DF outbreak, as these students usually have subjects regarding security and safety. For this reason, they might have more concerns about the safety of their surroundings compared to the business studies and science and engineering major students. A large number of the study population had DF, which corresponds to the enormous number of dengue cases in Dhaka [3,4,5,8,46]. Some students were also not convinced about the legitimacy of their infection, which indicates asymptomatic cases or cases without testing. Asymptomatic dengue cases have been prevalent across the countries over the years [1]. One study already claimed that the rapid expansion of dengue cases throughout Dhaka city and the whole country during the 2019 outbreak was due to the movement of massive asymptomatic dengue cases [3]. Besides, dengue infection has been historically prevalent among children and younger age groups [46,47,48] in Asia. Our study also disclosed that the Master’s-level students had more dengue infection than the first-year students from undergraduate level. The reason behind it is beyond the explanation of the current study. However, a previous study already revealed the increased seroprevalence of dengue virus among the older age group in Dhaka city [7].

### 4.2. Dengue Fever Knowledge, Attitudes, and Practices

Our findings have suggested that the university students in Dhaka city demonstrated a relatively better attitude than their knowledge and practices. The poignant impact of the COVID-19 pandemic might have a role in driving positive attitudes towards any infectious diseases. Nonetheless, these attitudes need to be translated into preventive measures. The participants likewise demonstrated exemplary knowledge scores about the fatal impact and common symptoms of DF. These findings correspond to various studies [8,28,49], contrary to another study conducted in an illiterate sample population [42]. It again confirms the prominence of education where the learner university students have more access to practical knowledge. There were challenges to distinguish between dengue infection and coronavirus infection during the pandemic. Mass testing facilities are also required for dengue cases to control the rapid outbreak during the pandemic. This study identified that the university students had poor knowledge about dengue virus transmission to the fetus during pregnancy [50,51,52,53]. Similar results were found in a previous study conducted among Malaysian people [28]. Interestingly, a significant number of the study population failed to recognize DF as an infectious disease, and one person can have DF more than once. It aligns with a previous study where it was mentioned that second-time DF could be severe [54]. Many of them could not correctly identify the *Aedes* mosquito breeding sites and the transmission cycle of DF virus (human-mosquito-human). It also corresponds to another study conducted on Malaysian university students [24]. Our findings urged for the DF campaign program along with the regular dengue surveillance activities. Web-based education and mobile applications can be effective tools for university students who normally have access to the Internet. Social media can also play a substantial role in this aspect [8]. University authorities may collaborate with the local and national authority or vice versa to disseminate the authentic knowledge to this hub group of community.

This study also demonstrates that students were concerned about the comprehensive approach for effective dengue surveillance, such as cleaning mosquito breeding sites. They strongly agreed to visit the doctor for their family member after showing any DF symptom. Similar results of comprehensive approach requirements were mentioned in previous studies [28,49,55]. Students agreed that the chemical fogging is not enough to control the DF outbreak, which supports another study [24], as fogging has some negative impact [56,57]. Numerous participants were not strongly interested in participating in the DF control public activity, which should be taken seriously to motivate these students towards different DF control campaigns.

### 4.3. Determinants of KAP Level

This study revealed the significant factors, such as gender, residential unit type, university year, and major field of study, associated with their KAP domain level in univariate analyses. However, multiple analyses identified only the residential unit and major as significant predictors of preventive practices and overall KAP level. Female students had better DF attitudes and practices compared to that of their male counterparts. This finding can align with the Bangladeshi social context, where a female typically seeks more safety than a male [17]. It also urges the campaign activities to motivate the male residents. Studies have already found a higher prevalence of DF among males than the female group [4,5,46,58,59]. Our findings suggest that, despite the high DF exposure of mixed-unit-residing students (different people and business activities are conducted here), they did not have sufficient knowledge and overall KAP compared to the high-rise- and low-rise-unit-dwelling students. Residential units should have the authority to conduct and monitor dengue surveillance activities. They can also perform awareness programs among their residents, and university students can be a pivotal part of this program. The first-year undergraduate students had better attitudes than the Master’s level students. The possible explanation could be that the fresher students might be more serious about their health and safety than their senior fellows. Senior students might be more confident in their knowledge level that they were careless regarding good attitude level. It indicates the distressed situation about these senior students since they were found to be more greatly infected than the first-year students (Table 1). Both univariate and multiple analyses confirmed again the urgency of dengue relevant subject and demonstration among different-majored university students, particularly the students from security and strategic studies. It should be noteworthy that an effective systematic approach is required to integrate infectious disease-related subjects and relevant demonstrations to prepare this learner group. First-year students could have theoretical understanding, where senior students could follow the practical demonstration. These students should also be assisted for further implementation of their learning in the community. In this way, long-term strategic planning involving university students could reduce the DF burden for the whole country.

### 4.4. Limitation of the Study

This study has some limitations. It was based on a self-reported online survey, which may have some bias. We used a convenience sampling technique due to the ongoing COVID-19 pandemic. We performed no further interventions following data collection. For example, students rated their living place as safe or risky in terms of DF, but this was not substantiated by direct observation because of the self-reported survey pattern and ethical concerns. For the same reason, we did not verify any clinical diagnosis record on their history of DF infection. Nonetheless, our exploratory study revealed several significant findings that might benefit university administrators, health and social professionals, as well as national and local Dhaka municipal authorities. In addition, it paves the way for future research by providing a large sample size and additional essential data.

## 5. Conclusions

In Bangladesh, Dhaka city has become the center of the DF outbreaks. This survey found a strong positive attitude about DF among university students residing in Dhaka. However, a large portion of the sample population lacked adequate awareness and preventative behaviors. Numerous students regarded their living conditions to be risky in the event of a DF breakout. Students from various disciplines are expected to take infectious disease-related courses and demonstrate their knowledge to be prepared for a future DF outbreak. Additionally, this might help lower the danger of DF spreading across the community since these young groups could share their knowledge with their family members and neighbors. Despite their high exposure and impacted status, the male group was shown to have negative attitudes and behaviors compared to their female counterparts. Mixed-unit occupants are often at a higher risk of developing DF because of their increased exposure and susceptibility. However, this study reveals that students living in high-rise and low-rise units have more knowledge and an overall higher KAP level than students residing in mixed units. Local and national authorities can enhance their dengue-monitoring efforts by launching an efficient campaign to convey accurate information to this population. Collaboration between the community and the universities may allow these students to prepare for future DF outbreaks. Authorities may be able to assist in this regard. It might also prepare the entire community via university students, the country’s future leaders.

## Figures and Tables

**Table 1:** Association of socio-demographic and academic information with living place’s safety and dengue infection history among university students of Dhaka city, Bangladesh.

Characteristics	(*n* (%))	Living Place’s Safety Perception against Dengue Outbreak (Mean ± SD ^1^)	Dengue Infection History (Mean ± SD ^1^)
1. Gender		*p* = 0.504 ^a^	*p* = 0.296 ^a^
− **Male**	306 (48.96)	3.23 ± 0.92	0.21 ± 0.40
− **Female**	319 (51.04)	3.16 ± 0.88	0.18 ± 0.38
2. Living with Family		*p* = 0.237 ^a^	*p* = 0.446 ^a^
− **Yes**	504 (80.64)	3.21 ± 0.90	0.20 ± 0.39
− **No**	121 (19.36)	3.12 ± 0.91	0.18 ± 0.38
3. Location in Dhaka City		*p* = 0.127	*p* = 0.235 ^a^
− **Dhaka South City** − **Corporation**	221 (35.36)	3.10 ± 0.84	0.22 ± 0.41
− **Dhaka North City** − **Corporation**	404 (64.64)	3.24 ± 0.93	0.18 ± 0.38
4. Residential Unit		*p* = 0.083 ^b^	*p* = 0.466 ^b^
− **High-Rise (>5-storey)**	340 (54.40)	3.20 ± 0.91	0.21 ± 0.40
− **Low-Rise (<=5-storey)**	252 (40.32)	3.24 ± 0.87	0.18 ± 0.38
− **Mixed**	33 (5.28)	2.79 ± 0.96	0.26 ± 0.44
5. University Type		*p* = 0.084 ^a^	*p* = 0.854 ^a^
− **Public**	540 (86.40)	3.16 ± 0.91	0.20 ± 0.39
− **Private**	85 (13.60)	3.36 ± 0.81	0.19 ± 0.39
6. University Year		*p* = 0.133	*p* * = 0.035 ^b^
− **1st**	125 (20.00)	3.07 ± 0.94	0.15 ± 0.35
− **2nd**	154 (24.64)	3.30 ± 0.83	0.18 ± 0.38
− **3rd**	209 (33.44)	3.21 ± 0.96	0.22 ± 0.41
− **4th**	107 (17.12)	3.22 ± 0.87	0.18 ± 0.38
− **Master’s**	30 (4.80)	2.93 ± 0.58/	0.38 ± 0.49
7. Major		*p* *** = 0.000 ^b^	*p* = 0.286 ^b^
− **Arts and Social Sciences**	143 (22.88)	3.20 ± 0.88	0.15 ± 0.35
− **Business Studies**	135 (21.60)	3.36 ± 0.92	0.25 ± 0.43
− **Security and Strategic Studies**	121 (19.36)	2.93 ± 0.89	0.21 ± 0.41
− **Science and Engineering**	129 (20.64)	3.35 ± 0.88	0.20 ± 0.39
− **Others (Miscellaneous)**	97 (15.52)	3.07 ± 0.87	0.18 ± 0.36
8. Dengue-Relevant Subject in Curriculum		*p* = 0.536 ^b^	*p* = 0.882 ^b^
− **Yes**	86 (13.76)	3.24 ± 0.94	0.19 ± 0.38
− **No**	379 (60.64)	3.16 ± 0.90	0.20 ± 0.39
− **Maybe**	160 (25.60)	3.25 ± 0.88	0.21 ± 0.40

* *p* < 0.05; *** *p* < 0.001. ^1^ SD, Standard Deviation; ^a^, Mann–Whitney U test; ^b^**,** Kruskal–Wallis test.

**Table 2:** Knowledge regarding dengue fever among university students of Dhaka city, Bangladesh.

Statements	Correct Response (n (%))	Incorrect Response (n (%))
Dengue is an infectious disease	381 (60.96)	244 (39.04)
Dengue fever can cause death	582 (93.12)	43 (6.88)
Common Symptoms of dengue infection are rash, headache, high fever, joint pain, muscle pain, nausea	547 (87.52)	78 (12.48)
*Aedes* mosquito type transmits dengue virus	556 (88.96)	69 (11.04)
*Aedes* mosquito has stripes on the body	508 (81.28)	117 (18.72)
**Dengue virus can be transmitted through direct contact with an infected person**	412 (65.92)	213 (34.08)
*Aedes* mosquitoes breeding site	339 (54.24)	286 (45.76)
*Aedes* mosquito can breed both indoors and outdoors	477 (76.32)	148 (23.68)
*Aedes* mosquito normally bites early in the morning and late evening	463 (74.08)	142 (22.72)
Dengue virus can be transmitted from infected pregnant mother to fetus	323 (51.68)	302 (48.32)
Person can be infected with dengue virus more than once	465 (74.40)	160 (25.60)
Dengue infection can be reduced by keeping surrounding areas clean and destroying potential breeding sites	570 (91.20)	55 (8.80)

Bold item shows the incorrect statement.

**Table 3:** Attitude towards dengue fever among university students of Dhaka city, Bangladesh.

Statements	* SA (*n* (%))	* A (*n* (%))	* N (*n* (%))	* DA (*n* (%))	* SDA (*n* (%))
I have the responsibility to ensure no *Aedes* eggs and/or larvae are in my house area	304 (48.64)	278 (44.48)	34 (5.44)	08 (1.28)	1 (0.16)
We should clean *Aedes* mosquito breeding sites, like water containers, storage tank, and plant pots one to three times a week	359 (57.44)	236 (37.76)	24 (3.84)	03 (0.48)	3 (0.48)
Authorities should demolish the potential breeding sites; chemical fogging alone is not enough to control dengue fever	364 (58.24)	228 (36.48)	27 (4.32)	05 (0.80)	1 (0.16)
We should regularly check the dengue situation around our area	323 (51.68)	258 (41.28)	26 (4.16)	16 (2.56)	2 (0.32)
Removal of mosquito breeding sites should be on a regular basis even in the period when there is no fever	299 (47.84)	283 (45.28)	34 (5.44)	07 (1.12)	2 (0.32)
Dengue fever control depends on the community commitment to remove mosquito breeding sites	307 (49.12)	268 (42.88)	37 (5.92)	10 (1.60)	3 (0.48)
I will take part in a dengue fever control public activity	213 (34.08)	306 (48.96)	96 (15.36)	06 (0.96)	4 (0.64)
I will bring my family member to see a doctor immediately if he/she has dengue fever symptoms	380 (60.80)	215 (34.40)	23 (3.68)	06 (0.96)	1 (0.16)

* SA, strongly agree; A, agree; N, neutral; DA, disagree; SDA, strongly disagree. Expected responses are in bold.

**Table 4:** Preventive practices towards dengue fever among university students of Dhaka city, Bangladesh.

Statements	Yes (*n* (%))	No (*n* (%))
I call the municipal authority for fogging	160 (25.60)	465 (74.40)
I use aerosol and/or liquid mosquito repellent and/or mosquito coil and/or electrical mosquito mat and/or mosquito bed net	572 (91.52)	53 (8.48)
I properly cover water containers used for water storage	548 (87.68)	77 (12.32)
I scrub and clean the inner sides of the containers	532 (85.12)	93 (14.88)
I check for the presence of *Aedes* eggs and/or larvae inside or outside the house	313 (50.08)	312 (49.92)
I keep the plant pots clear and drain the extra water	540 (86.40)	85 (13.60)
I visit the hospital for test and treatment when I see the symptoms of dengue fever	530 (84.80)	95 (15.20)
I follow the latest information from trusted sources, such as WHO or my local and national health authorities	494 (79.04)	131 (20.96)

**Table 5:** Correlation in KAP domain among university students of Dhaka city, Bangladesh.

Association	r-Value ^1^ (95% CI ^2^)	*p*-Value	Interpretation
Knowledge and Attitude	0.209	0.000 ***	Positive Correlation
Knowledge and Practice	0.211	0.000 ***	Positive Correlation
Attitude and Practice	0.210	0.000 ***	Positive Correlation

^1^ r, correlation coefficient; ^2^ CI, confidence intervals. *** p < 0.001.

**Table 6:** Univariate analysis predictors of knowledge, attitude, practices, and overall KAP level towards dengue fever among university students of Dhaka City, Bangladesh.

Characteristics	Knowledge		Attitude		Practice		Overall KAP	
	**OR ^1^ (95% CI ^2^)**	***p***	**OR (95% CI)**	***p***	**OR (95% CI)**	***p***	**OR (95% CI)**	***p***
1. Gender								
− **Male**	1		1		1		1	
− **Female**	1.09 (0.78–1.53)	0.591	1.74 (1.04–2.96)	0.036 *	1.43 (1.02–2.00)	0.038 *	1.07 (0.74–1.56)	0.702
2. Living with Family								
− **Yes**	1.35 (0.89–2.03)	0.149	0.70 (0.33–1.37)	0.333	1.08 (0.70–1.64)	0.717	1.50 (0.96–2.32)	0.073
− **No**	1		1		1		1	
3. Location in Dhaka City								
− **Dhaka South City Corporation**	0.74 (0.53–1.05)	0.094	1.13 (0.67–1.97)	0.648	0.91 (0.64–1.29)	0.591	1.00 (0.68–1.47)	0.997
− **Dhaka North City Corporation**	1		1		1		1	
4. Residential Unit								
− **High-Rise (>5-storey)**	4.79 (2.30–10.41)	0.000 ***	2.42 (0.91–5.75)	0.056	1.58 (0.75–3.26)	0.216	3.76 (1.80–7.86)	0.000 ***
− **Low-Rise (<=5-storey)**	2.84 (1.36–6.21)	0.006 **	2.34 (0.87–5.69)	0.072	1.71 (0.80–3.56)	0.157	2.82 (1.34–5.94)	0.006 **
− **Mixed**	1		1		1		1	
5. University Type								
− **Public**	1.32 (0.82–2.11)	0.244	0.85 (0.36–1.75)	0.675	0.88 (0.53–1.44)	0.629	0.86 (0.48–1.46)	0.584
− **Private**	1		1		1		1	
6. University Year								
− **1st**	1.61 (0.68–3.71)	0.265	4.96 (1.44–17.16)	0.009 **	1.06 (0.44–2.44)	0.888	2.25 (0.87–5.54)	0.083
− **2nd**	1.07 (0.46–2.39)	0.867	2.50 (0.81–6.91)	0.087	1.25 (0.52–2.83)	0.600	1.31 (0.53–3.03)	0.541
− **3rd**	0.93 (0.41–2.03)	0.865	1.43 (0.50–3.61)	0.466	0.95 (0.41–2.10)	0.904	1.04 (0.43–2.34)	0.927
− **4th**	1.48 (0.62–3.46)	0.363	2.42 (0.76–7.22)	0.116	1.17 (0.48–2.74)	0.719	1.98 (0.76–4.94)	0.146
− **Master’s**	1		1		1		1	
7. Major								
− **Others (Miscellaneous)**	1		1		1		1	
− **Arts and Social Sciences**	0.76 (0.42–1.36)	0.367	0.85 (0.30–2.19)	0.741	0.79 (0.42–1.45)	0.454	0.44 (0.20–0.91)	0.031 *
− **Business Studies**	0.40 (0.22–0.70)	0.002 **	0.32 (0.12–0.75)	0.013 *	0.52 (0.28–0.93)	0.031 *	0.25 (0.12–0.51)	0.000 ***
− **Security and Strategic Studies**	0.57 (0.31–1.03)	0.067	0.70 (0.25–1.83)	0.484	0.29 (0.16–0.52)	0.000 ***	0.27 (0.12–0.54)	0.000 ***
− **Science and Engineering**	0.85 (0.46–1.54)	0.594	0.92 (0.32–2.50)	0.880	0.77 (0.41–1.43)	0.415	0.70 (0.31–1.51)	0.369
8. Dengue-Relevant Subject in Curriculum								
− **Yes**	1.71 (1.02–2.98)	0.049 *	0.54 (0.28–1.08)	0.070	1.61 (0.96–2.81)	0.079	1.16 (0.67–2.08)	0.597
− **Maybe**	1.15 (0.78–1.71)	0.487	0.88 (0.49–1.66)	0.688	1.25 (0.84–1.88)	0.266	1.22 (0.79–1.92)	0.376
− **No**	1		1		1		1	
9. Dengue Infection History								
− **Yes**	1.45 (0.94–2.31)	0.102	0.69 (0.39–1.31)	0.238	0.79 (0.52–1.22)	0.285	1.30 (0.80–2.18)	0.309
− **Maybe**	0.52 (0.18–1.56)	0.235	0.66 (0.17–4.32)	0.594	1.11 (0.36–4.10)	0.861	0.57 (0.19–1.89)	0.324
− **No**	1		1		1		1	

* *p* < 0.05; ** *p* < 0.01; *** *p* < 0.001. ^1^ OR, odds ratio; ^2^ CI, confidence intervals.

**Table 7:** Multiple analysis predictors of preventive practices and overall KAP level towards dengue fever among university students of Dhaka City, Bangladesh.

Characteristics	Practice		Overall KAP	
	**aOR ^1^ (95% CI ^2^)**	***p***	**aOR (95% CI)**	***p***
1. Gender				
− **Male**				
− **Female**	1.41 (0.99–2.01)	0.057		
2. Living with Family				
− **Yes**			1.33 (0.83–2.11)	0.233
− **No**				
3. Location in Dhaka City				
− **Dhaka South City Corporation**				
− **Dhaka North City Corporation**				
4. Residential Unit				
− **High-Rise (>5-storey)**	1.56 (0.72–3.28)	0.248	3.38 (1.56–7.31)	0.001 **
− **Low-Rise (<=5-storey)**	1.80 (0.82–3.91)	0.136	2.63 (1.20–5.75)	0.014 *
− **Mixed**				
5. University Year				
− **1st**			2.56 (0.94–6.73)	0.059
− **2nd**			1.74 (0.66–4.30)	0.243
− **3rd**			1.39 (0.54–3.34)	0.927
− **4th**			2.64 (0.95–7.00)	0.470
− **Master’s**				
6. Major				
− **Others (Miscellaneous)**				
− **Arts and Social Sciences**	0.73 (0.38–1.36)	0.327	0.42 (0.19–0.87)	0.025 *
− **Business Studies**	0.55 (0.29–0.99)	0.0526	0.24 (0.11–0.54)	0.000 ***
− **Security and Strategic Studies**	0.31 (0.16–0.56)	0.000 ***	0.26 (0.12–0.54)	0.000 ***
− **Science and Engineering**	0.76 (0.40–1.40)	0.381	0.64 (0.28–1.42)	0.283
7. Dengue Relevant Subject in Curriculum				
− **Yes**	1.32 (0.75–2.39)	0.347		
− **Maybe**	1.07 (070–1.64)	0.757		
− **No**				

* *p* < 0.05; ** *p* < 0.01; *** *p* < 0.001. ^1^ aOR, adjusted odds ratio; ^2^ CI, confidence intervals.

## Data Availability

The data presented in this study are available on request from the corresponding author.

## Supplementary Materials

The following are available online at https://www.mdpi.com/article/10.3390/ijerph19074023/s1, File S1: Knowledge, Attitude, and Practices towards Dengue Fever among University Students of Dhaka city, Bangladesh.
