# Supplementary material for: Knowledge, Attitude, and Practices towards Dengue Fever among University Students of Dhaka City, Bangladesh
Source: Int J Environ Res Public Health. 2022 Mar 28;19(7):4023. doi: 10.3390/ijerph19074023 (PMC8998586; doi:10.3390/ijerph19074023)
Supplement: Supplementary file 1 [file ijerph-19-04023-s001.zip › ijerph-1467819-supplementary.pdf]

File S1. Knowledge, Attitude and Practices towards Dengue Fever among University Students of Dhaka city, Bangladesh

This online survey will take 8~10 minutes. Your Contribution will be highly appreciated!
In this survey you will be asked some questions to evaluate personal measures against dengue. Please read the questions very well and provide your answers accordingly. Please note that all the information you provide us is totally CONFIDENTIAL and would be used only by the research team for the purposes of research. This research has been approved by the Department of Disaster and Human Security Management, Bangladesh University of Professionals maintaining all ethical issues. Please contact the research team for further query.

Socio-demographic Information

1. Gender

- Male
- Female

2. Are you living with Family?

- Yes
- No

3. Where do you live in Dhaka?

- Dhaka North City Corporation
- Dhaka South City Corporation

4. Type of your residential unit?

- Up to 5 story building
- More than 5 story building
- Mixed-use building (Shop, factory, office, and residence in the same building)

5. University type

- Public
- Private

6. Year

- 1st
- 2nd
- 3rd
- 4th
- Masters

7. What is your major?

- ○ Arts and Social Sciences
- ○ Business Studies
- ○ Science and Engineering
- ○ Security and Strategic Studies
- ○ Others (Miscellaneous)

8. Is there any Subject related to Dengue in University Curriculum?

- ○ Yes
- ○ No
- ○ Maybe

9. Have you had Dengue before?

- ○ Yes
- ○ No
- ○ Maybe

Knowledge

10. Do you know Dengue is an infectious disease?

- ○ Yes
- ○ No
- ○ Maybe

11. Do you know Dengue fever can cause death?

- ○ Yes
- ○ No
- ○ Maybe

12. Do you know the Common Symptoms of Dengue infection are Rash, Headache, High Fever, Joint Pain, Muscle Pain, Nausea?

- ○ Yes
- ○ No
- ○ Maybe

13. By which type of Aedes mosquito Dengue virus is transmitted?

- ○ Male
- ○ Female
- ○ I don't know

14. Does Aedes mosquito has stripes on the body?

- Yes
- No
- Maybe

15. Dengue virus can be transmitted through direct contact with an infected person

- True
- False
- Maybe

16. Do you know where Aedes mosquitoes breed?

- Clean and stagnant water
- Dirty Water and flowing drain
- I don't know

17. Aedes mosquito breed both indoor and outdoor?

- Yes
- No
- Maybe

18. Dengue Mosquito likes to bite early in the morning and late evening

- Yes
- No
- Maybe

19. Do you know Dengue virus can be transmitted from infected pregnant mother to fetus?

- Yes
- No
- Maybe

20. Do you know one person can be infected with Dengue virus more than once?

- Yes
- No
- Maybe

21. Do you think Dengue infection can be reduced by keeping you surrounding areas clean and destroying potential breeding sites?

- Yes
- No
- Maybe

Attitude

22. It is my responsibility to make sure there are no Aedes eggs and/or larvae in my house area

- Strongly Agree
- Agree
- Neutral
- Disagree
- Strongly Disagree

23. My family members and neighbors should clean Aedes mosquito breeding site like water containers, storage tank, Plant pot one to three times a week.

- Strongly Agree
- Agree
- Neutral
- Disagree
- Strongly Disagree

24. Only chemical fogging by the authority is not enough to prevent dengue infection, authority should also demolish the potential breeding sites

- Strongly Agree
- Agree
- Neutral
- Disagree
- Strongly Disagree

25. We should check dengue situation or hotspots around our area regularly

- Strongly Agree
- Agree
- Neutral
- Disagree
- Strongly Disagree

26. It is necessary to continue the removal of mosquito breeding sites even during the period when there is no outbreak.

- Strongly Agree
- Agree
- Neutral
- Disagree
- Strongly Disagree

27. Dengue outbreak in my community can be controlled if every household is committed to

remove mosquito breeding

- Strongly Agree
- Agree
- Neutral
- Disagree
- Strongly Disagree

28. I will take part in a public activity for dengue control or removal of mosquito breeding sites

- Strongly Agree
- Agree
- Neutral
- Disagree
- Strongly Disagree

29. If my family member has symptom of dengue fever, I will bring him/her to see a doctor for immediate treatment

- Strongly Agree
- Agree
- Neutral
- Disagree
- Strongly Disagree

Practice

30. Do you Call Municipality authority for fogging?

- Yes
- No

31. Do you Use aerosol and/or liquid mosquito repellent and/or mosquito coil and/or electrical mosquito mat and/or mosquito bed net?

- Yes
- No

32. Do you properly cover Water containers used for water storage?

- Yes
- No

33. Do you scrub and clean the inner sides of the containers?

- Yes
- No

34. Do you Check for the presence of Aedes eggs and/or larvae inside or outside the house?
   - Yes
   - No

35. Do you keep the plant pots clear and drain the extra water?
   - Yes
   - No

36. Do you go to hospital for test and treatment when you see the symptoms of Dengue?
   - Yes
   - No

37. Do you follow the latest information from trusted sources, such as WHO or your local and national health authorities?
   - Yes
   - No
